# Predication of hub target genes of differentially expressed microRNAs contributing to *Helicobacter pylori* infection in gastric non-cancerous tissue 

**Published:** 2019

**Authors:** Mohsen Norouzinia, Mona Zamanian Azodi, Diba Najafgholizadeh Seyfi, Ali Kardan, Ali Naseh, Zahra Akbari

**Affiliations:** 1 *Gastroenterology and Liver Diseases Research Center, Research Institute for Gastroenterology and Liver Diseases, Shahid Beheshti University of Medical Sciences, Tehran, Iran *; 2 *Proteomics Research Center, Shahid Beheshti University of Medical Sciences, Tehran, Iran*; 3 *Tehran Medical Branch, Islamic Azad University, Tehran, Iran*; 4 *Pediatric and Neonatal Ward, Taleghani Hospital, Shahid Beheshti University of Medical Sciences, Tehran, Iran*; 5 *Laser Application in Medical Sciences Research Center, Shahid Beheshti University of Medical Sciences, Tehran, Iran*

**Keywords:** MicroRNA, Helicobacter pylori, Regulatory network, Target genes, Hubs, Functional analysis

## Abstract

**Aim:**

The main goal of this investigation was to provide an overview on *H.pylori* effect on gastric tissue via bioinformatics analysis of microarray-identified miRNAs and its target genes.

**Background:**

MicroRNAs which control about 30 to 60% of gene expression in human body play a critical role in different cell growth stages. Expression modification of non-coding (NC) RNAs in H.pylori infections requires further investigations to provide better understanding of their roles in the body.

**Methods:**

GSE54397, the microRNA microarray dataset, was analyzed by GEO2R, the online GEO database for detection of differentially expressed microRNAs and lastly the potential target genes as well as their associated pathways.

**Results:**

A total of 244 miRNAs were detected as differentially expressed (p<0.05 and FC>2) in non-cancerous tissue of gastric with H.pylori infection in comparison with tissues without H.pylori infection. The findings indicated that hub microRNAs and target genes of up-regulated network are KIF9, DCTN3, and CA5BP1 along with hsa-miR-519d, hsa-miR-573, hsa-miR-646, hsa-miR-92a-1, hsa-miR-186, and hsa-miR-892a, respectively. For the down-regulated network, genes of RABGAP1, HSPB11 and microRNAs of hsa-miR-620, hsa-miR-19b-2, hsa-miR-555, and hsa-let-7f-2 were hubs. Most of the up-regulated microRNAs are involved in gastric cancer development while there is no evidence for the down-regulated ones. Yet, all of the hub down-regulated miRNAs are reported to have associations with different kinds of cancer.

**Conclusion:**

The introduced hub miRNAs and genes may serve as feasible markers in the mechanisms of *H.pylori* infection for different kinds of gastric diseases, in particular gastric cancer. However, their role requires further investigations.

## Introduction

 Expression modification of non-coding (NC) RNAs in infection by *H.pylori* has not been widely studied (1). MicroRNAs as one of the main controllers for about 30 to 60% of gene expression in human body are key participants in cell different stages of growth (2). These are recognized as RNAs class with 18 to 25 nucleotide sequences that are post-transcript modifiers (3). They can silence the target mRNAs expression by binding to their 3′-UTR region (4). Their role has been proved in many diseases such as cancer (5). 

Meanwhile, *Helicobacter pylori* as a mobile, microaerophilic, Gram-negative bacteria is recognized and thought of as one of the key reasons behind gastric cancer and various gastric diseases, specifically, a number of benign, pre-malignant, and malignant lesions developed in the digestive system (6, 7). It is suggested that H. pylori infection is the most notable risk factor for the advancements of noncardia gastric cancers (8). In most instances of gastric cancers, H. pylori is the main reason behind the underlying gastritis, which can boost gastric carcinogenesis, usually through atrophilic gastritis, intestinal metaplasia as well as dysplasia (9). Studies also illustrate a potential connection that links H. pylori infection and H. pylori associated gastric diseases to colorectal neoplasia, though further investigation and research is required to fully elucidate this association (10).

Molecular studies could be helpful to provide knowledge of new conditions caused by environmental influence such as infection status. H.pylori as gram-positive bacteria could induce different conditions in the human body. The molecular mechanism by which this procedure occurs remained to be investigated. One way is high throughput study of differentially expressed molecules in a condition with H.pylori infection. In this regard, differentially expressed either genes or miRNAs could be detected through comparison of healthy condition and infection with the bacteria. Furthermore, the identified differentially expressed agents could be more analyzed through bioinformatics as a complementary study (11). Bioinformatics study such as regulatory network analysis is promising in this regard for discovering the most promising targeting Differentially Expressed (DE)-microRNAs (12). In this sense, this study tries to identify the key microRNAs and target genes involved in the mechanisms underlying the H.pylori pathogenesis in the human body by screening the corresponding regulatory networks. 

## Methods

This work is based on identification of differentially expressed microRNAs or DE-miRNAs. The dataset chosen for our study was the GEO Accession number of GSE54397 under the platform of GPL15159 (Agilent-031181 Unrestricted_Human_miRNA_V16.0_Microarray 030840) available on Gene Expression Omnibus (GEO) database. GEO2R (http://www.ncbi.nlm.nih.gov/geo/geo2r/), R-based which is a GEO online tool analyzed the differentially expressed microRNAs of the mentioned dataset. Specifically, two groups of samples from four groups were selected for this investigation. The first two groups were cancerous tissue HP+ and HP- while the other two groups were noncancerous regions HP+ and HP-, 32 samples altogether. This online tool analyzes DE-miRNAs by performing statistical analysis of t-test or analysis of variance. This dataset was provided by a study entitled “microRNA expressions in gastric cancer” by Chang H, et.al in 2014 on 16 patients diagnosed with gastric adenocarcinoma. These patients were age, sex, and H*. pylori* infection matched. The biopsy was carried out at Seoul National University Bundang Hospital and the end the samples was used for microarray study. 

The 250 identified DE-miRNAs were ranked based on significance (p>0.05) of differential expressions in 8 noncancerous samples with *H.pylori* compared to 8 noncancerous samples without *H.pylori* infection. These none-coding RNAs were used for further analysis in terms of interaction properties. To this aim, the miRNAs that were significant (p<0.05) and their fold change was above 2 (FC>2) were chosen. 

ClueGO+CluePedia, the Cytoscape 3.7.2 plug-in (13), analyzes the differentially expressed miRNAs in terms of target genes associations (14, 15). The criterion assigned for this procedure was detecting genes from miRanda source (miRNA-V5-2012-07-19.txt.gz) with a kappa score or miRanda-SCORE-v5≥ 0.6 and designating 10 hub genes. Furthermore, annotation from Pathway, KEGG, and Wikipathway of target genes of both regulatory networks of up-regulated and down-regulated networks was done by ClueGO. Kappa score≥ 0.4 as the default score, gene per term: 1, and gene Percentage per term: 1% were considered. 

## Results

**Figure 1:**
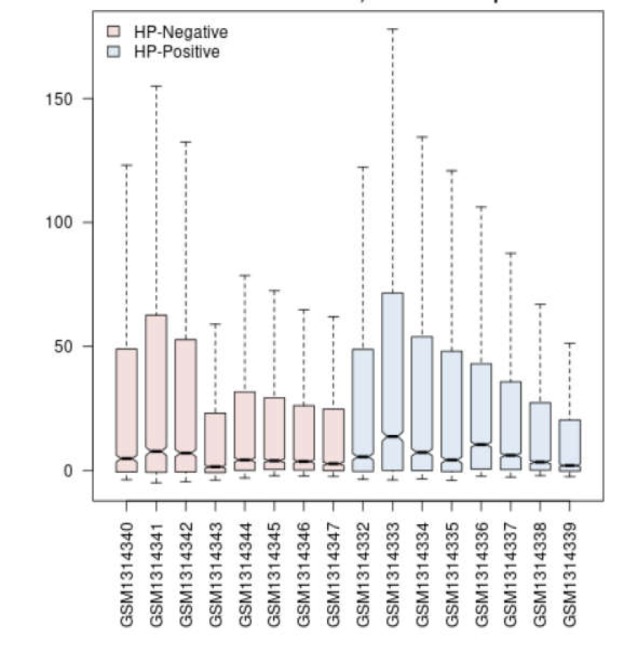
A comparison of gene expression profiles of two groups of 16 samples of non-cancerous HP negative (pink) and HP positive (blue). The x-axis and y-axis indicate the range of expression and biological replications for both groups, respectively

In order to compare two groups of interest in terms of differential expressions, initially the statistical evaluation of groups of samples was handled by box plot analysis to assess whether these groups are eligible or not in this regard (Figure 1). 

**Table 1:** The list of target genes of up-regulated miRNAs detected by miRanda source, CluePedia. (Kappa Score:0.6). The asterisked miRNAs are among the hub microRNAs

R	Target Gene	Degree	Highest Kappa Score	Highest scored miRNA
1	KIF9	20	0.8	hsa-miR-377
2	DCTN3	16	0.8	hsa-miR-612
3	CA5BP1	16	0.8	hsa-miR-593
4	ADRA1A	15	0.7	hsa-miR-892a*
5	C1orf43	15	0.8	hsa-miR-367
6	DCBLD1	15	0.8	hsa-miR-302b
7	MRPL43	15	0.8	hsa-miR-519d*
8	CREB3L4	15	0.8	hsa-miR-573*
9	SARDH	14	0.7	hsa-miR-646*
10	CBWD5	14	0.7	hsa-miR-7-2

The box plot analysis indicated that the values are median-centered and the data are appropriate for further investigations. The GEO2R analysis indicated 250 top rated statistically significant DE-miRNAs in which 244 miRNA were identified by p<0.05 and fold change (FC)>2. These dysregulated DE- miRNAs differentiate non-cancerous tissues infected with *H.pylori* in comparison with HP negative samples. Among them, 124 ones were up-regulated while 120 were down-regulated in the infected subjects. The list of DE-miRNAs is available as supplementary in Table 1. Two regulatory networks were obtained for up-regulated and down-regulated genes. Specifically, to identify targeted genes of the DE-miRNAs, a regulatory network via CluePedia was constructed (the data not shown). In this analysis, miRNAs are prioritized for interaction based on connection to hub genes (Kappa Score: 0.6). 

**Figure 2:**
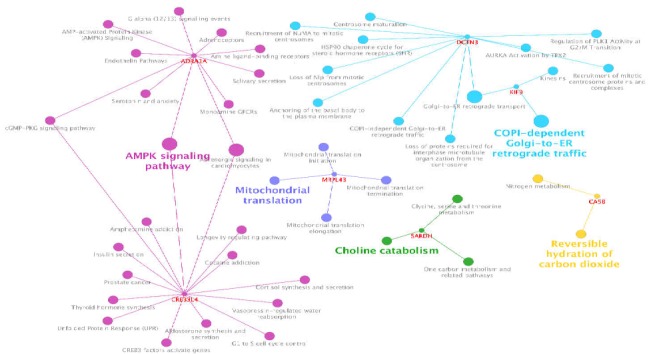
Pathway analysis of hub target genes of up-regulated miRNAs via ClueGO. Kappa Score ≥ 0.5 is considered. Names of clusters are highlighted and the other terms are backgrounded

**Figure 3:**
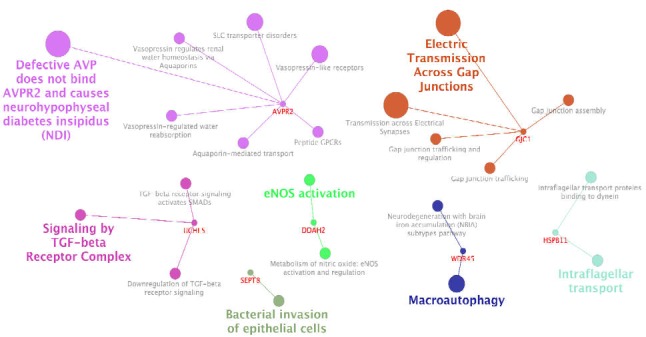
Pathway analysis of hub target genes of down-regulated miRNAs via ClueGO. Kappa Score ≥ 0.4 is considered. Names of clusters are highlighted and the other terms are backgrounded

**Table 2:** The list of target genes of down-regulated miRNAs detected by miRanda source, CluePedia. Kappa Score:0.6 was considered for analysis

R	Target Gene	Degree	Highest Kappa Score	Highest scored miRNA
1	RABGAP1	16	0.8	hsa-miR-599
2	HSPB11	15	0.7	hsa-miR-26a-2
3	WDR45	14	0.7	hsa-miR-19b-2*
4	CBWD1	14	0.8	hsa-miR-590-3p
5	GJC1	13	0.8	hsa-miR-137
6	SEP-8	13	0.7	hsa-miR-578
7	DDAH2	13	0.7	hsa-miR-328
8	UCHL5	13	0.7	hsa-miR-590-3p
9	AVPR2	13	0.7	hsa-miR-485-5p
10	TM2D1	13	0.8	hsa-miR-607

Among 124 up-regulated miRNAs, 115 ones were found by microRNA source where 63 miRNAs were connected to the 10 target genes via 154 interactions. The other 52 ones remained isolated. The list of target hub genes and Degree of hub genes, highest interacting miRanda score (Kappa score) and the highest scored interacting miRNA to these hubs are tabulated in Table 1.

 In addition, pathway analysis of the target genes describes more properties of their biological contributions as shown in Figure 2. 

KIF9 has had the highest degree of 20 in the up-regulated regulatory network and hsa-miR-377 has been the highest scored regulator. The top hubs are KIF9, DCTN3, and CA5BP1. On the other hand, hsa-miR-519d, hsa-miR-573, hsa-miR-646, hsa-miR-92a-1, hsa-miR-186, and hsa-miR-892a are the hub micorRNAs all with the degree of 5. Four of them assigned with star in Table 1 are among these hubs. 

AMPK signaling pathway, COPI-dependent Golgi-to-ER retrograde traffic, mitochondrial translation, Choline catabolism, and Reversible hydration of carbon dioxide are the highlighted groups for the hub target genes of the up-regulated regulatory network. 

The same procedure for downregulated DE-miRNAs was adopted to detect target genes as a regulatory network via CluePedia (the data not shown). Among 120 queried ones, 103 microRNAs including 53 connected and 50 isolated individuals were recognized. Likewise, miRNAs are prioritized for the interaction based on hub features of target genes with the details of hub genes properties presented in Table 2. Pathway analysis for the target genes also indicates more features of their biological contributions (see Figure 3). 

RABGAP1 has the highest degree of 16 in the down-regulated regulatory network, which is followed by HSPB11 in this category. On the other hand, hsa-miR-620, hsa-miR-19b-2, hsa-miR-555, and hsa-let-7f-2 are the hub microRNAs whose degree for the first two has been 6 and for the last two five, with hsa-miR-19b-2 in Table 2 being among the hubs. 

The down-regulated pathways include “Defective AVP does not bind AVPR2 and causes neurohypophyseal diabetes insipidus (NDI)”, “Electric Transmission across Gap Junctions”, “Signaling by TGF-beta Receptor Complex”, “Bacterial invasion of epithelial cells”, “Macroautophagy”, and “Intraflagellar transport”. They are the proposed group terms for down-regulated regulatory network.

## Discussion

In view of the fact that molecular analysis could provide further knowledge of pathogenicity activity of bacteria such as H. pylori in the human, a bioinformatics approach in this regard has been conducted. A GEO data of DE-miRNAs in tissues with H. Pylori infection in comparison with tissues without this infection was applied for further analysis. In this study, DE-miRNAs with statistically significant values were selected for regulatory network and functional assessments. At first, DE-microRNAs were assigned by GEO2R analysis and then the differentially expressed ones with criteria of the fold change of ≥ 2 and p-value ≤ 0.05 were selected. Most of the DE-miRNAs are significant for the fold change of at least two or above this threshold. This result suggests that H.pylori has a substantial impact on molecular changes of normal tissues. The candidate microRNAs were then evaluated for regulatory networks of up-regulation and down-regulation. The two networks were concluded using ClueGO+CluePedia as subnetworks. The subnetworks were those with significant interactions with the hub genes. In the first regulatory network, about half of the up-regulated miRNAs were paired with the hub genes. The highest degree hub microRNAs and target genes were identified through regulatory network screening. These included hsa-miR-519d, hsa-miR-573, hsa-miR-646, hsa-miR-92a-1, hsa-miR-186, and hsa-miR-892a as hub microRNAs as well as KIF9, DCTN3, and CA5BP1 as target genes.

The introduced hub target genes were then assessed for functional annotations properties which could be important in the mechanisms of H.pylori influence. Among the identified pathways, AMPK signaling pathway was the leading group for the up-regulated network. The same procedure was done for down-regulated miRNAs. The detected microRNAs and target genes as hubs were hsa-miR-620, hsa-miR-19b-2, hsa-miR-555, and hsa-let-7f-2, as well as RABGAP1 and HSPB11, respectively. The pathway of Defective AVP does not bind AVPR2 causing neurohypophyseal diabetes insipidus (NDI); it is the most highlighted group term for down-regulated network. These Pathways (up-regulated and down-regulated) could be the targets of the differential expressing miRNA because of their relevance to hub genes both for up-regulated and down-regulated ones. Literature review of the introduced elements could provide more information in this regard. Six microRNAs as referred earlier were identified as dysregulated hubs. Among them, four miRNAs have been previously reported for gastric cancer. hsa-miR-519d has been reported to have associations with precancerous lesions in gastric cancer pathogenicity (16). hsa-miR-646 is the other hub down-regulated in gastric cancer. Its low level is associated with cancer development (17). This miRNA was both up- and downregulated in the current analysis; however, while its down-regulation is dominant, it is not considered as a hub in the down-regulated network. hsa-miR-92a-1-5p is also important as a diagnostic biomarker in gastric cancer (18). The downregulation of miR-186 and its connection with Twist1 and HIF-1α has been recognized as antitumor growth biomarker in gastric cancer. Its relationship with Twist1 is important in cancer high stage and the size of tumor. In other words, it contributes to cell growth process and its expression could have therapeutic values (19, 20). Conversely, one of the hub elements of the down-regulated network has shown linkage with gastric diseases based on reviewing previous studies; however, they have shown associations with other types of cancer (21-24). This indicates that these hub down-regulated microRNAs could also be potentially involved in gastric cancer trigger. Overall, these agents could be important in the onset of any kind of gastric disease associated with H.Pylori, infections and especially gastric cancer. Nonetheless, their associations are yet to be established. 

The regulatory correlation between the 10 hub DE-miRNAs and 5 related target genes as an integrated study may indicate that the identified hub miRNAs and genes could be important in the mechanism of helicobacter pylori pathogenesis.
